# Genetic integrity is still maintained in natural populations of the indigenous wild apple species *Malus sylvestris* (Mill.) in Saxony as demonstrated with nuclear SSR and chloroplast DNA markers

**DOI:** 10.1002/ece3.6818

**Published:** 2020-09-28

**Authors:** Stefanie Reim, Frank Lochschmidt, Anke Proft, Monika Höfer

**Affiliations:** ^1^ Federal Research Centre for Cultivated Plants Institute for Breeding Research on Fruit Crops Julius Kühn Institute (JKI) Dresden Germany; ^2^ Green League Osterzgebirge e.V. Dippoldiswalde Germany

**Keywords:** chloroplast DNA, European wild apple, haplotypes, hybridization, microsatellites, species delimitation

## Abstract

*Malus sylvestris* (Mill.) is the only indigenous wild apple species in Central Europe. Agriculture, forestry, and urbanization increasingly endanger *Malus sylvestris* natural habitats. In addition, the risks of cross‐hybridization associated with increase in the cultivation of the domesticated apple *Malus × domestica* (Borkh.) threaten the genetic integrity of *M. sylvestris*. The present study investigated the number of hybrids, genetic diversity, and genetic structure of 292 putative *M. sylvestris* that originate from five different natural *M. sylvestris* populations in Saxony, Germany. All samples were genetically analyzed using nine nuclear microsatellite markers (ncSSR) and four maternally inherited chloroplast markers (cpDNA) along with 56 apple cultivars commonly cultivated in Saxony. Eighty‐seven percent of the wild apple accessions were identified as pure *M. sylvestris*. The cpDNA analysis showed six private haplotypes for *M. sylvestris,* whereas three haplotypes were present in *M. sylvestris* and *M. × domestica*. The analysis of molecular variance (AMOVA) resulted in a moderate (ncSSR) and great (cpDNA) variation among pure *M. sylvestris* and *M. × domestica* individuals indicating a low gene flow between both species. The genetic diversity within the pure *M. sylvestris* populations was high with a weak genetic structure between the *M. sylvestris* populations indicating an unrestricted genetic exchange between these *M. sylvestris* populations. The clear distinguishing of *M. sylvestris* and *M. ×domestica* confirms our expectation of the existence of pure *M. sylvestris* accessions in this area and supports the argument for the implementation of preservation measures to protect the *M. sylvestris* populations in Saxony.

## Graphical abstract

The present study investigated the number of hybrids, genetic diversity, and genetic structure of 292 putative *M. sylvestris* that originate from five different natural populations in Saxony, Germany. Genetic analysis were performed with nuclear and chloroplast markers. We detected a high genetic diversity within the pure *M. sylvestris* populations but with a weak genetic structure between the *M. sylvestris* populations indicating an unrestricted genetic exchange between these *M. sylvestris* populations.

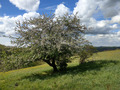

## 1 INTRODUCTION

The genus *Malus* Mill. comprises 25–47 species, depending on the taxonomic classification (Robinson et al., 2001). While several *Malus* species are indigenous to Asia, *Malus sylvestris* (Mill.) is the only indigenous wild apple species in Central Europe. The spatial distribution of this European crabapple ranges from South Scandinavia to the Iberian Peninsula and from the Volga to the British Isles (Robinson et al., 2001). Despite this vast distribution area, *M. sylvestris* is very rarely seen and in fact endangered in most European countries (Bitz et al., 2019; Coart et al., 2006; Höltken et al., 2014; Larsen et al., 2006; Reim et al., 2012, 2013; Wagner et al., 2014). Its economical insignificance and the present‐day intensive land usage by forestry and agriculture are the main reasons why wild apple trees have been displaced into areas with more or less unfavorable growing conditions. However, *M. sylvestris* trees have, except for their light requirements, low demands on environmental conditions. They are therefore able to survive in such niche areas. *M. sylvestris* populations are often very small and spatially distributed, which may lead to a reproductive isolation of individual populations (Reim et al., 2017; Ruhsam et al., 2019; Wagner et al., 2014). Most *Malus* species are self‐incompatible and need compatible pollination partners to maintain the natural regeneration of the populations (Hanke et al., 2020). Insects such as bees or bumblebees mainly distribute apple pollen, and most pollination events occur in a short distance (50–100 m) around the pollinator. However, if sufficient pollination partners are missing, the distance of pollination can remarkably increase (Reim et al., 2017). Since no reproductive barriers prevent the hybridization between different species within the genus *Malus*, the probability of admixture with domesticated apples (*Malus* × *domestica* Borkh.) increases particularly in small populations (Korban, 1986; Larsen et al., 2006; Reim et al., 2017). Such hybridization events endanger the genetic integrity of *M. sylvestris* by replacing pure *M. sylvestris* trees with hybrids (Allendorf et al., 2001). Thus, the conservation of genetic resources must extend to include the preservation of pure *M. sylvestris* trees.

Knowledge of population genetic diversity and genetic structures is crucial in the identification of pure genotypes, which is integral in studies for the implementation of sustainable conservation strategies (Castiglione et al., 2010). However, in closely related species such as in *Malus* a clear distinguishing of species remains a challenge. Delimitation of *M. sylvestris* and *M*. ×*domestica* is particularly difficult because of their close relationship (Duan et al., 2017).

Several morphological characteristics were described as useful for the classification of *M. sylvestris* like hairiness of leaf lower surface, hairiness of the flower stem; fruit size, and cover color (Wagner, 1996). However, the main disadvantage of morphological traits is their high level of variation depending on various environmental factors. For example, the intensity of hairiness, which functions as protection against evaporation, can vary during the growing season. Another example is the intensity of over color, which depends on exposure to sun light. Therefore, genetic markers are an indispensable tool for taxonomical studies of species. In conservation genetic studies, microsatellite markers or simple sequence repeats (SSRs) are still the markers of choice (Bitz et al., 2019; Coelho et al., 2018; Kaczmarczyk, 2019; Lea et al., 2018) and are useful tools for a putative identification of “hybrids” within potential wild apple populations (Koopman et al., 2007; Larsen et al., 2006). For closely related species, such as the species within the genus *Malus*, noncoding DNA regions of the chloroplast genome could also be an additional useful genetic tool to study the relationship among species (Fan et al., 2019; Khadivi‐Khub et al., 2014; Tang et al., 2014; Volk et al., 2015). Because of their slow rate of molecular evolution and the conserved structure of their genome, chloroplast markers were developed for several important plant species (Heinze, 2007). In *Malus,* several intergenic spacer and introns regions in the chloroplast genome were identified and from these regions, primers successfully generated and applied for phylogenetic studies (Coart et al., 2006; Fan et al., 2019; Khadivi‐Khub et al., 2014; Tang et al., 2014; Volk et al., 2015; Xu et al., 2019).

In our study, *M. sylvestris* accessions from five natural populations in Saxony (Germany) were genetically investigated using nine nuclear microsatellites (ncSSR) and four chloroplast DNA (cpDNA) markers. All putative *M. sylvestris* individuals were analyzed with ncSSR markers in order to identify pure accessions. Pure *M*. *sylvestris* accessions and 56 *M. × domestica* genotypes were further analyzed with four cpDNA markers to ascertain haplotype variation within and between these species. We further estimated the genetic diversity within the *M. sylvestris* populations and the genetic structure among these populations in the study area. The results of this study are the basis for the implementation of conservation measures.

## 2 MATERIALS AND METHODS

### 2.1 Study site, sampling, and DNA isolation

**Figure 1:**
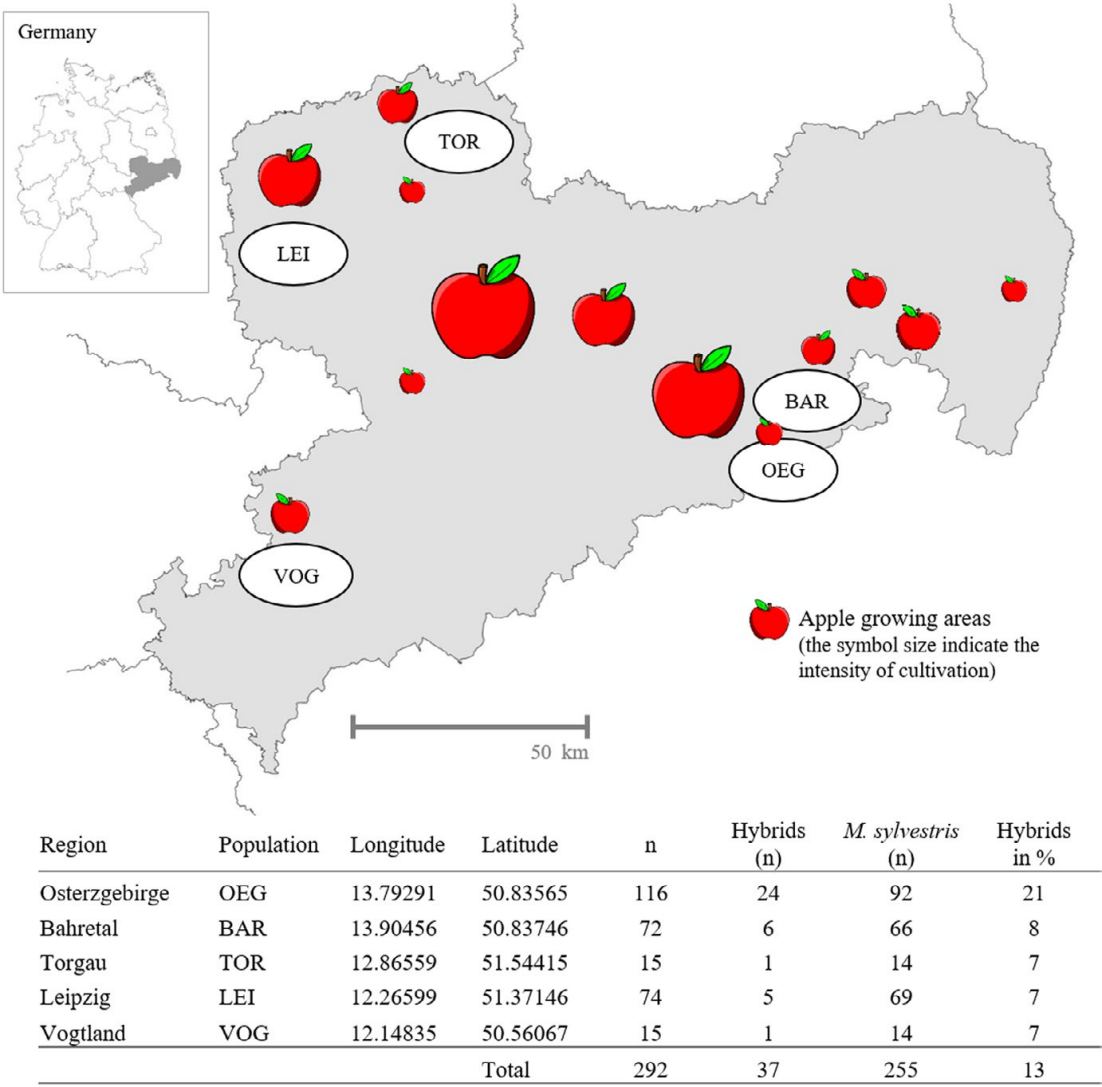
Origin, number of sampled *M. sylvestris* trees and number of hybrids in five natural populations in Saxony, Germany

The study was carried out in the federal state of Saxony, in Southeast Germany. Saxony comprises an area of 18,413 square kilometers, and hills and mountains mainly compose the landscape. Two hundred and ninety‐two potential *M. sylvestris* trees sampled from five different areas within Saxony (Figure 1) formed the basis of this study. The mapped trees were mainly found in sparse forests, along forest edges, stonewalls, or other open landscape structures. The tree density within the populations was low in most cases with partly large distances between the single trees. The trees of some population such as in the East Ore Mountains (OEG) were distributed over several square meters. Compact populations with more than 50 individuals in a close spatial network only existed in the Leipziger Auwald (LEI) and Bahretal (BAR). Seven percent of the sampled trees were located in a nature reserve. The remaining 93% were located in unprotected areas. The selection of trees was based on several morphological characters described by Wagner (1996) including fruit diameter <3.5 cm, no fruit over color, and absence of hairiness in the leaf lower surface, for example. In addition, 56 apple cultivars (*M. × domestica* Borkh.) often cultivated in Germany were included as control genotypes. The location of the captured *M. sylvestris* trees and the names of the 56 cultivars are available in Dryad at https://doi.org/10.5061/dryad.g79cnp5n2. Leaf material of these 56 apple cultivars was provided by the Fruit Genebank of the Institute of Breeding Research on Fruit Crops of the Julius Kühn‐Institut (JKI) at Dresden‐Pillnitz.

Fresh leaf materials were collected in 2‐ml tubes and dried using silica gel according to a modified protocol of (Chase & Hills, 1991). Leaf materials were stored at room temperature until DNA isolation. LGC Genomics (Berlin, Germany) performed DNA isolation and quantification using the semi‐automated high‐throughput Genespin™ platform with the company proprietary Kleargene™ extraction chemistry.

### 2.2 SSR and cpDNA analysis

The genetic analysis was performed at the state‐owned enterprise Sachsenforst in Pirna. Nine ncSSR primer pairs (CH01F02, CH01F03b, CH01H01, CH01H10, CH02C09, CH04C07, GD12, GD147, Hi02C07) developed for *M*. × *domestica* (Liebhard et al., 2002) were combined in three multiplexes with three primers per multiplex (Table S1). Detailed information about the molecular characteristics of these markers is available at http://hidras.unimi.it/HiDRAS‐SSRdb/pages/index.php.

Four cpDNA primer pairs located at the *matK_dup* (NCBI GenBank: AF309231), *rps16* intron (NCBI GenBank: JQ391664) region and the intergenic spacer region *rps16_trnQ* (NCBI GenBank: AB604694) and *rpl2_trnH* (NCBI GenBank: JQ392141), respectively, were developed based on *Malus* sequence data obtained from the NCBI database (www.ncbi.nlm.nih.gov; Table 1).

**Table 1:** Chloroplast DNA (cpDNA) and nuclear SSR (ncSSR) markers used in this study. Table 1: Chloroplast DNA (cpDNA) and nuclear SSR (ncSSR) markers used in this study

	Primer sequence forward	Primer sequence reverse	Fragment size (bp)
Chloroplast region			
*rps16_trnQ*	CAGTAAGTACCATTCGCTTTTTATC	TTTCGACCAGTCTTCCGTTT	304–312
*rpl2_trnH*	CACTTAACACAAAAGCAGAAAAAGA	GGATCAAGGCAGTGGATTGT	147–170
*matK_dup*	GGTTATGCGATCGTAGAAATGG	TTCCTTCCCTATACACGACTCT	158–178
*Rps16_Intron*	GACAAAAAGGGTTAGAGACCACTC	CTCGTACGGCTCGAGAAAAT	245–249
ncSSR marker			
*CH01f02*	ACCACATTAGAGCAGTTGAGG	CTGGTTTGTTTTCCTCCAGC	153–227
*CH01f03b*	GAGAAGCAAATGCAAAACCC	CTCCCCGGCTCCTATTCTAC	139–183
*CH01h01*	GAAAGACTTGCAGTGGGAGC	GGAGTGGGTTTGAGAAGGTT	114–134
*CH02c09*	TTATGTACCAACTTTGCTAACCTC	AGAAGCAGCAGAGGAGGATG	233–257
*CH01h10*	TGCAAAGATAGGTAGATATATGCCA	AGGAGGGATTGTTTGTGCAC	094–114
*CH04c07*	GGCCTTCCATGTCTCAGAAG	CCTCATGCCCTCCACTAACA	098–135
*GD12*	TTGAGGTGTTTCTCCCATTGGA	CTAACGAAGCCGCCATTTCTTT	150–200
*GD147*	TCCCGCCATTTCTCTGC	GTTTAAACCGCTGCTGCTGAAC	135–155
*Hi02c07*	AGAGCTACGGGGATCCAAAT	GTTTAAGCATCCCGATTGAAAGG	108–149

Multiplex PCR was performed using the Type‐It kit (Qiagen, Germany) according to the manufacturer's protocol with three primer pairs per PCR in a total volume of 10 µl. PCR fragments were analyzed on a CEQ 8000XL Genetic Analyzer System (Beckman Coulter, Germany), for which forward primers were labeled with BMN‐5, BMN‐6, and DY751 (Biomers, Germany).

### 2.3 Hybridization and population structure analysis

First, samples were grouped into pure *M. sylvestris*, *M. × domestica* or hybrids using the model‐based clustering method with STRUCTURE software version 2.3.4. (Pritchard et al., 2000). In order to improve the accuracy of the inference, the analysis was performed with prior information on the population (POPINFO model) in which each individual was defined either as "*M. sylvestris*" or "*M*. × *domestica*." To accurately classify the individuals, 5 runs with *K* = 2 was performed with the program. The parameters were 50,000 burn‐in periods and 50,000 Markov Chain Monte Carlo repetitions using the admixture model with correlated allele models. The probability of membership to the *M. × domestica* genepool for the 56 reference *M*. ×*domestica* cultivars was > 90% indicating a low introgression from *M. sylvestris*. Conversely, a probability > 90% of membership to the *M. sylvestris* genepool was chosen as threshold for the assignment of pure *M. sylvestris* accessions. All *M. sylvestris* individuals with a probability of lower than 90% that the assigned population is right were classified as hybrid. Secondly, the number of natural *M. sylvestris* populations (*K*) was estimated with STRUCTURE. For this, we run the program again including all pure *M. sylvestris* accessions with *K* varying from 2 to 10 with 5 runs for each *K* value without POPINFO model. The remaining parameters were described as above. STRUCTURE HARVESTER (Earl & Vonholdt, 2012) was used for detecting the most likely value for *K* based on Evanno's ΔK method (Evanno et al., 2005). To examine the genetic structure of *M. sylvestris*, a principal coordinate analysis (PCoA) was performed based on the distance matrix data set of the 255 pure *M. sylvestris* accessions and the 56 reference apple cultivars in GeneAlex ver. 6.5 with 1,000 random permutations.

### 2.4 Diversity analysis

The mean number of alleles by locus (*N_a_*), effective number of alleles (*N_e_*), observed heterozygosity (*H_o_*), expected heterozygosity (*H_e_*), and number of private alleles (*PA*) were calculated for each nuclear SSR loci within the pure *M. sylvestris* individuals and the apple cultivars using the software GENALEX ver. 6.5 (Peakall & Smouse, 2006, 2012). Allelic richness (*Ar*) was calculated with the software ADZE (Szpiech et al., 2008) using the rarefaction method to correct differences in population sizes (Kalinowki, 2005). Because of the small sample size of the TOR and VOG populations, the *Ar* was weighted to fourteen individuals.

The allele frequencies of the chloroplast DNA markers were compared among the *M. sylvestris* individuals and the apple cultivars.

An "analysis of molecular variance" (AMOVA) was performed based on nuclear and chloroplast marker data. The molecular variance (*ɸ*_PT,_ an analogue of *F*_st_) and the migration rate (*N_m_*) were calculated among the pure *M. sylvestris* genotypes and the apple cultivars as well as among the different *M. sylvestris* populations using GENALEX ver. 6.5.

The correspondence (*rxy*) between geographic and genetic distance was performed by Mantel test with statistical testing by 9,999 permutations using the software GENALEX ver. 6.5 (Mantel, 1967).

## 3 RESULTS

### 3.1 Genetic differentiation between *M. sylvestris* and *M. × domestica*

**Figure 2:**
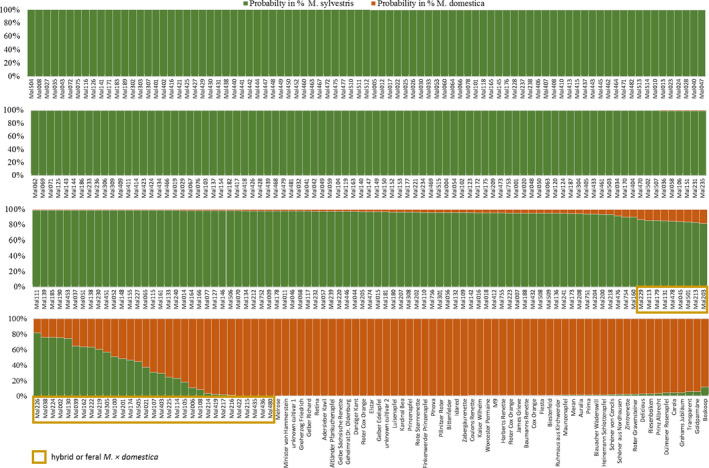
Barplot indicating the genetic affiliation of each individual to either *M. sylvestris* or *M. ×domestica*

Of 292 putative *M. sylvestris,* 255 (87%) were assigned as pure *M. sylvestris* species. The remaining 37 individuals were assigned as hybrids or feral *M*. ×*domestica* (Figure 2).

The four cpDNA markers showed allelic patterns with 12 different alleles with two to four alleles per chloroplast region. Of the 12 different alleles, seven were present in both *M. sylvestris* and *M* × *domestica* samples while five alleles were species specific (Figure S1).

**Figure 3:**
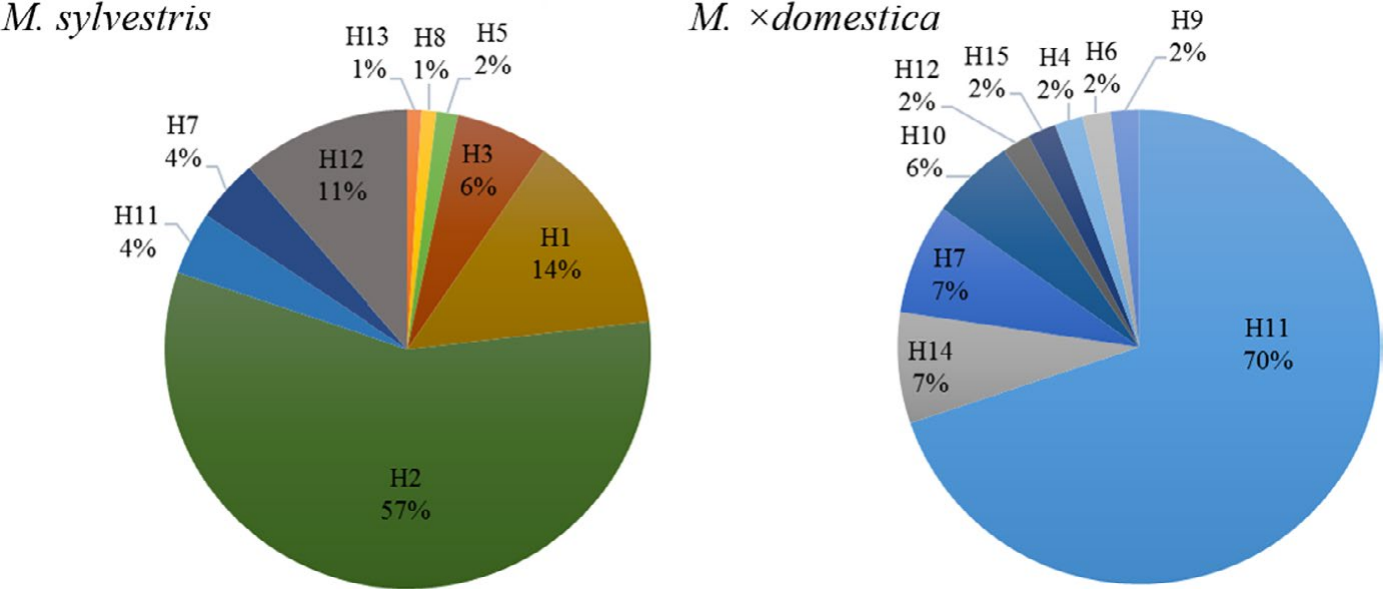
Frequency of the chloroplast 15 haplotype for *M. sylvestris* and *M. × domestica*

The combination of these different alleles resulted in 15 different haplotypes (Table S1) (Figure 3). In *M*. ×*domestica*, the most frequent haplotype was H11, which was observed for 70.0% of genotypes. The most frequent haplotype within the pure *M. sylvestris* accessions was H2 (57.3%). Twelve haplotypes were not observed for both species: H1, H2, H3, H5, H8, and H13 were not observed in the investigated apple cultivars whereas H4, H6, H9, H10, H14, and H15 were not found in the analyzed *M. sylvestris* genotypes. Only three haplotypes (H7, H711, and H12) were present in both *M. sylvestris* and *M. × domestica*.

**Figure 4:**
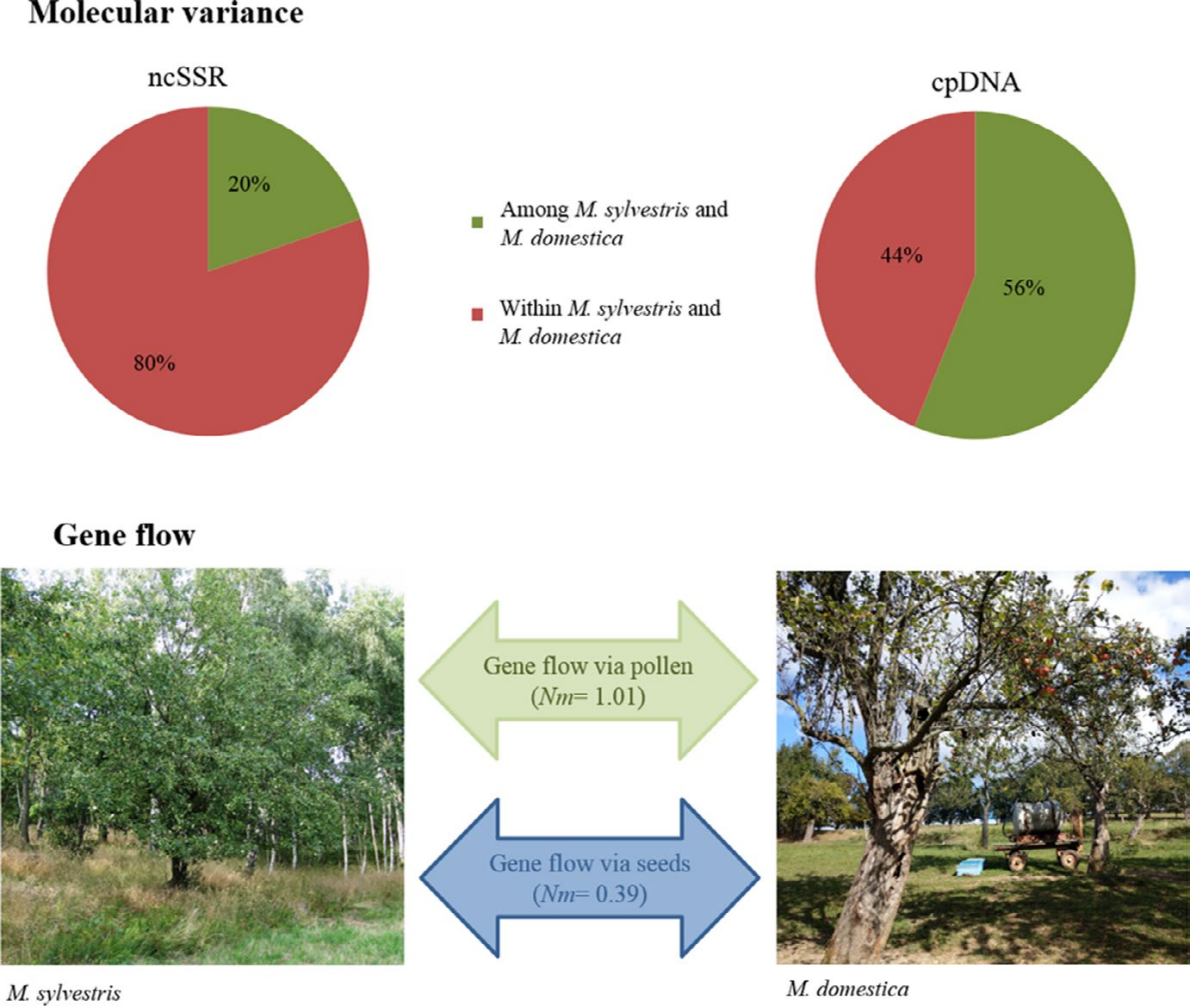
Molecular variance and estimated gene flow based on ncSSR and cpDNA data between wild apples and apple cultivars

The analysis of molecular variance (AMOVA) showed significant results for the differentiation between *M. sylvestris* and *M. ×domestica* (Figure 4). Based on the ncSSR, a moderate variation of 20% among the species was calculated (*ɸ*_PT_ = 0.20 *p* = .001). The effective gene flow was about one migrant per generation (*N_m_ = *1.01). Based on cpDNA data, a great variation of 56% among pure *M. sylvestris* and the apple cultivars (*ɸ*_PT_ = 0.56 *p* = .001) was detected. The effective gene flow was much less than one successful migrant per generation (*N_m_ = *0.39).

### 3.2 High genetic diversity and weak genetic structure between the *M. sylvestris* populations

All ncSSR markers showed reproducible results with one or two amplified fragments per genotype. High genetic diversity was observed for the pure *M. sylvestris* genotypes with an average number of alleles of *N_a_* = 10, an average effective number of alleles of *N_e_* = 4, a mean expected heterozygosity of *H_e_* = 0.73, and a mean allelic richness of *Ar* = 5.03 (Table 2). These diversity parameters were only slightly lower for *M. sylvestris* than for the *M*. ×*domestica* reference cultivars, indicating a still high level of genetic diversity in the populations investigated. The highest level of genetic diversity was found in the both southeastern Population OEG and BAR with *H_e_* = 0.78. Despite the large number of individuals, the LEI population showed the lowest genetic diversity with *H_e_* = 0.68. Only one private allele with a frequency > 0.05 was found in the TOR population, and all other populations had no private alleles.

**Table 2:** Number of alleles, number of effective alleles, allelic richness, observed and expected heterozygosity in five different *M. sylvestris* populations and the *M. × domestica* reference cultivars

Population	*N*	*N_a_*	*N_e_*	*H_o_*	*H*_e_	*PA*	*Ar*
BAR	66	12	5	0.74	0.78	0.00	5.45
LEI	68	12	4	0.65	0.68	0.00	4.48
OEG	89	14	5	0.76	0.78	0.00	5.69
TOR	14	8	4	0.71	0.72	0.00	5.22
VOG	14	6	4	0.69	0.69	0.00	4.33
mean		10	4	0.71	0.73	0.00	5.03
*M. × domestica*	53	13	5	0.79	0.79	5.00	5.65

Note

*N:* number of analyzed individuals; *N_a_*: number of different alleles; *N_e_*: number of effective alleles (=1/(∑*p_i_* ^2^)); *p_i_*: relative frequency of the *i*th allele; *PA:* number of private alleles with a frequency >5%, *Ar*: Allelic richness, weighted to fourteen individuals.

**Figure 5:**
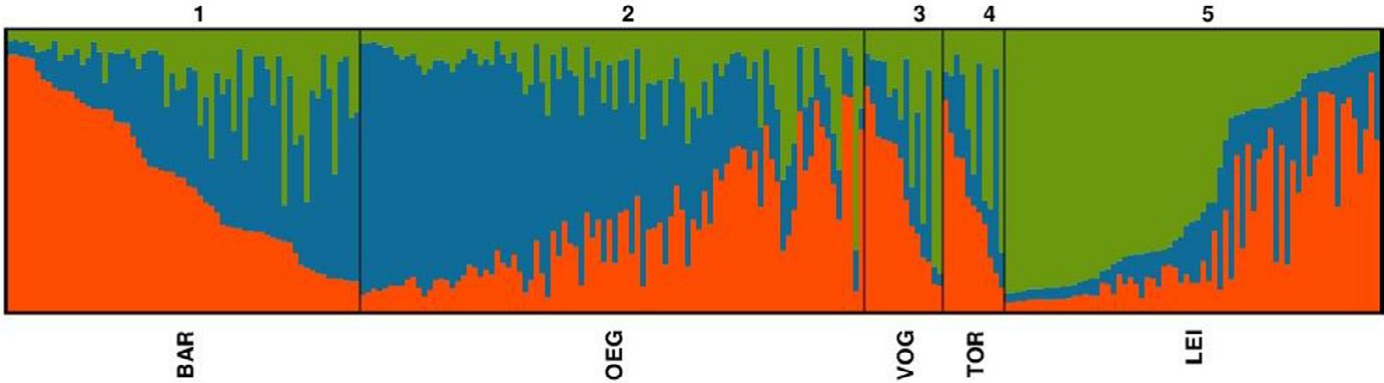
Cluster assignment (*K* = 3) of the examined *M. sylvestris* populations (*N* = 255, 5 population in Saxony) after model‐based clustering using STRUCTURE. Every vertical bar represents a single accession, the different colors indicate the assignment to each genetic cluster: orange= cluster 1; blue= cluster 2; green= cluster 3.

After analysis of the STRUCTURE output with STRUCTURE HARVESTER based on the Evanno method, the most likely number of genetic groups was *K* = 3 (Earl & Vonholdt, 2012; Evanno et al., 2005). Therefore, the individuals of the five *M. sylvestris* population were grouped into three genetic clusters indicated by three different colors in Figure 5. No population showed individuals with allele frequencies specific to only one genetic cluster. Nevertheless, alleles of genetic cluster 1 (orange) were more frequent in the BAR, VOG, and TOR population, whereas alleles of cluster 2 (blue) were more frequent in OEG. Alleles of cluster 3 (green) were mainly attributed to the LEI population. These results indicate only weak population structures between the Saxon *M. sylvestris* populations.

**Figure 6:**
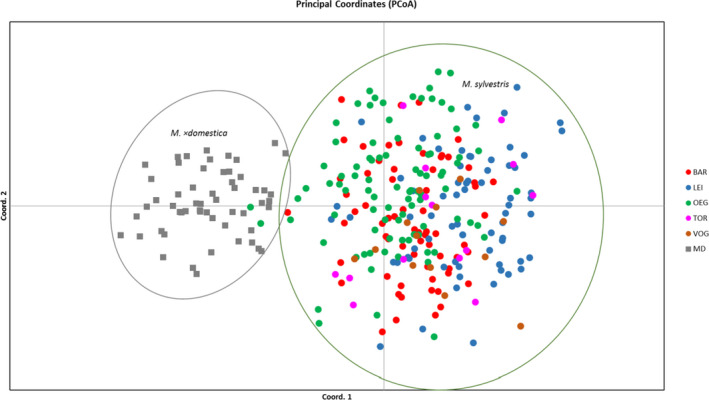
Principal coordinate analysis (PCoA) of pairwise distances between *M. sylvestris* accessions and the fifty‐six reference apple cultivars calculated on ncSSR markers. The *M. sylvestris* populations are indicated by different circle colors; red: Bahretal (BAR), blue: Leipzig (LEI), green: Osterzgebirge (OEG), pink: Torgau (TOR), brown: Vogtland (VOG), gray quadrat: *M. ×domestica* (MD).

In general, PCoA supported the results of the STRUCTURE analysis and showed low genetic differences between the *M. sylvestris* accessions of the five population investigated in Saxony (Figure 6). However, two accessions of *M. sylvestris* (green symbols), previously identified by STRUCTURE as pure *M. sylvestris* accessions, showed a very close relationship to *M*. × *domestica*, indicating that these accessions also hybrids.

**Figure 7:**
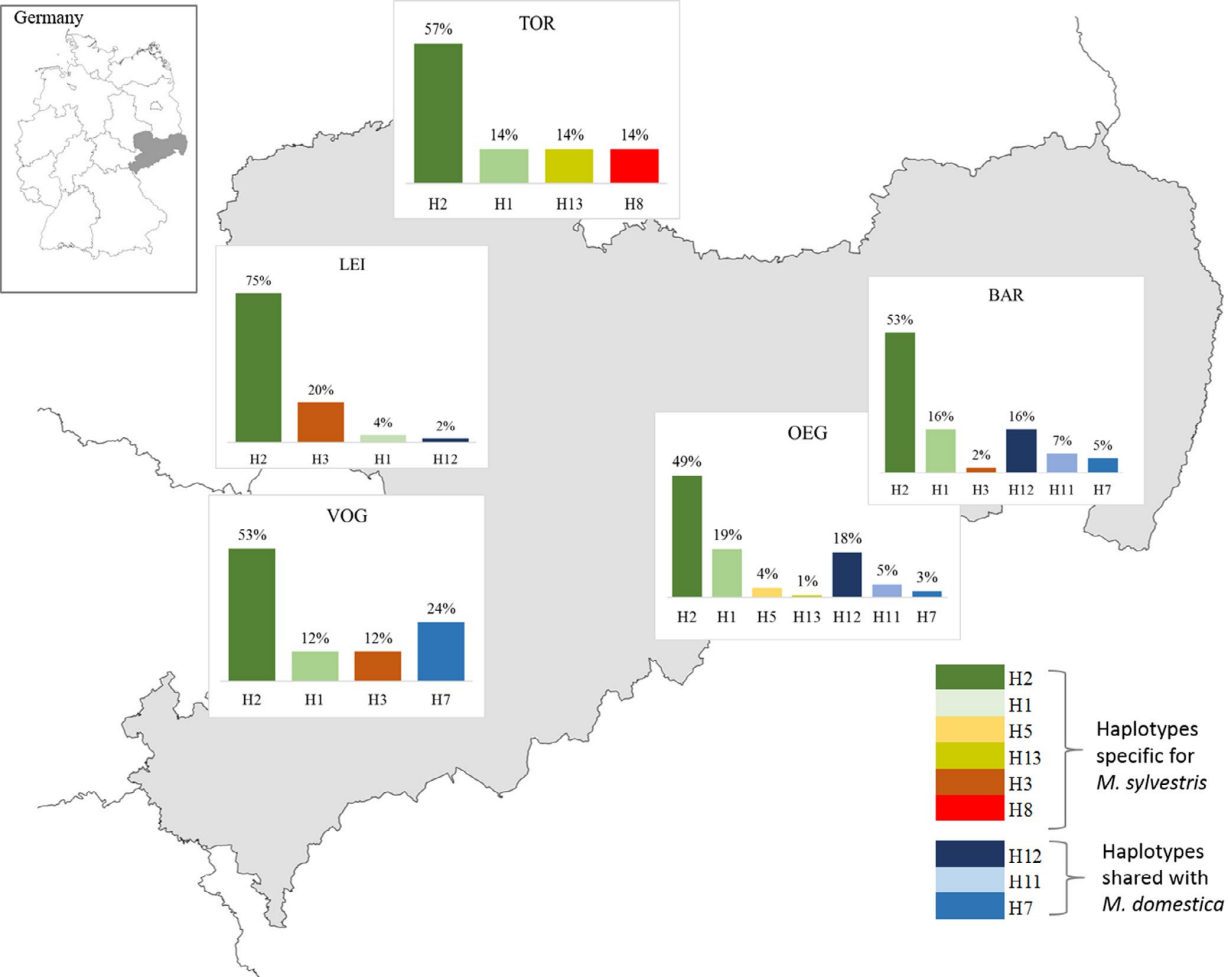
Haplotype variation in the five different pure *M. sylvestris* populations in Saxony

Based on the cpDNA data, also weak population structures were observed (Figure 7). Of the nine different haplotypes, H2 and H1 were detected in all *M. sylvestris* populations with different frequencies in each population. Haplotype H3 was found in the populations LEI, VOG, and BAR, which are located in the western and eastern parts of Saxony. H13 was found in the southeastern population OEG and the northern population TOR. The both haplotypes H5 and H8 were the only private haplotypes and observed only in OEG and TOR, respectively.

At least one of the haplotypes shared with *M. × domestica* (H7, H11, and H12) were also detected in the *M. sylvestris* populations with one exception. The population TOR showed only *M. sylvestris*‐specific haplotypes (H2, H1, H13, and H8). In the both southeastern populations OEG and BAR, all three *M. ×domestica* shared haplotypes were observed with the highest frequency for H12 (18% and 16%, respectively) (Figure 7). In the population VOG, only the *M. ×domestica* shared haplotype H7 was detected, but with a high frequency of 24%. The western population LEI showed only H12 as shared haplotype with a low frequency of 2%.

After AMOVA, the differences between the five *M. sylvestris* populations were significant, but low (Table 3). Based on ncSSR, the variation among the *M. sylvestris* populations was 6% and cpDNA variation was only slightly higher with 7%. The number of migrants among the *M. sylvestris* populations was high with *N_m_* = 4.14 (ncSSR) and *N_m_* = 7.07 (cpDNA) indicating random mating among each population and weak genetic structure of the populations. Mantel test resulted in a significant (*p* = .01) but low correlation coefficient *rxy* = 0.05 suggesting that the geographic distance had hardly any effect on the genetic variance in the Saxony wild apple populations.

**Table 3:** Genetic variation (AMOVA) among and within *M. sylvestris* populations based on ncSSR and cpDNA data

	Variation	ɸ_PT_	*p*	*N_m_*
Based on ncSSR data				
Among *M. sylvestris* populations	6%	0.06	.001	4.14^a^
Within *M. sylvestris* populations	94%			
Based on cpDNA data				
Among *M. sylvestris* populations	7%	0.07	.001	7.07^b^
Within *M. sylvestris* populations	93%			

Note

*ɸ*_PT_ = Estimated variation among populations/(Estimated variation within populations + Estimated variation among populations).

^a^ *N_m_* = number of migrants per generation [(1/*ɸ_PT_*) − 1]/4.

^b^ *N_m_* = number of migrants per generation [(1/*ɸ*_PT_) − 1]/2.

## 4 DISCUSSION

### 4.1 Identification of pure *Malus sylvestris* individuals

Knowledge of the genetic integrity of *M. sylvestris* and the clear identification of hybrids is one of the important prerequisites for the implementation of sustainable conservation measurements and the maintenance of pure species in natural populations. However, a clear distinguish between the European crabapple and cultivated apples is difficult because of molecular similarity between both species (Coart et al., 2006). Nevertheless, newer and more sensitive marker systems such as SNPs may be suitable for a clear differentiation of both species.

Some studies clearly demonstrated introgression of *M. sylvestris* DNA into the nuclear genome of *M*. ×*domestica* (Cornille et al., 2012; Harrison & Harrison, 2011). The contribution of *M. sylvestris* into the *M*. ×*domestica* gene pool can be very high (e.g., for the German cultivar “Landsberger Reinette” up to 48%) but depends strongly on the cultivar (Cornille et al., 2012). In our study, all 56 M*. × domestica* cultivars showed a low introgression from *M. sylvestris* (> 90% genetic information belong to the *M. × domestica* genepool). Based on this, all *M. sylvestris* samples and apple cultivars were clearly assigned in either a "*M. sylvestris* cluster" or "*M. × domestica* cluster." Putative *M. sylvestris* individuals with admixture genetic information of more than 10% of *the M. × domestica* gene pool were identified as hybrid (13%). This discrimination threshold (*Q* > 0.90) was very strict compared to other studies (Coart et al., 2003; Höltken et al., 2014; Schnitzler et al., 2014), and therefore, the frequency of hybrids is slightly higher in our population but corresponds well to the morphological characteristics of the sampled *M. sylvestris* accessions. These results indicate that a genetic differentiation of both species based on ncSSR is still possible although nuclear genome markers disclose less genetic differences in closely related species (Korotkova et al., 2014; Zheng et al., 2014).

### 4.2 Haplotype variation between *M. sylvestris* and *M*. × *domestica*

In closely related species such as apple, the investigation of phylogenetic relationships based on different cell genomes is recommended (Nikiforova et al., 2013). Chloroplast DNA is maternally inherited and hybridization events are limited to gene flow by seeds which results in a higher level of genetic differentiation among species or populations (Ennos, 1994; Korotkova et al., 2014; Shizuka et al., 2015).

In our study, 20% of the detected haplotypes were common in *M. sylvestris* and *M. × domestica* suggesting that these haplotypes are not species specific. Cross‐species shared chloroplast haplotypes were observed for several perennial species such as birch, eucalyptus and willows (Fogelqvist et al., 2015; Nevill et al., 2014; Palme et al., 2004). Within the genus *Malus*, shared haplotypes were also identified in studies including numerous apple individuals of different cultivars and species (Savolainen et al., 1995; Volk et al., 2015). *M. × domestica* is known to be an admixed species from a number of progenitor species such as *M. sieversii, M. orientalis, M. sylvestris,* and *M. prunifolia* (Cornille et al., 2013; Robinson et al., 2001; Velasco et al., 2010; Volk et al., 2015). Thus, shared haplotypes may reflect the historical introgression between different *Malus* species due to gene flow or retention of ancestral polymorphisms (Koopman et al., 2007; Nevill et al., 2014; Volk et al., 2015).

Nevertheless, despite the historical admixture within the genus *Malus*, species‐specific haplotypes exist as shown in our study in which 80% of the *M. sylvestris* haplotypes were absent in the 56 M*. × domestica* cultivars. However, that other apple cultivars not analyzed in this study could possess these haplotypes cannot be excluded. The high level of *M. sylvestris*‐specific haplotypes could be explained by genetic drift or selection. Descendants of *M. sylvestris* in our study site could have lost one or more of the identical haplotypes over time, supported by the absence or rarity of further introgression events. This assumption was confirmed by the low number of migrants per generation (*N_m_* = 0.39; cpDNA) in our study indicating a low historical gene flow through seed dispersal between *M*. × *domestica* and *M. sylvestris*. Evolutionary studies showed that large fruit development in domesticated crops such as *M. ×domestica* correlates with a reduction in seed number/ fruit and a loss in natural seed‐dispersal mechanism (Spengler, 2019). This would also explain the remarkable haplotype variations between *M. sylvestris* and *M. × domestica*.

The results of the AMOVA also affirmed that numerous *M. sylvestris* individuals were hardly affected by gene flow by pollen in past times in our study area (*N_m_* = 1.01). A similar rarity of gene flow among *M. sylvestris* and *M. × domestica* in the Rhine Valley or in Finland was observed by Schnitzler et al. (2014) and Bitz et al. (2019) and was explained by the only partial overlap in flowering time of these both species. However, the frequency of hybrids varied between the different *M. sylvestris* populations in our study area. In particular, the OEG population was more affected from hybridization with *M. ×domestica* with 21% identified hybrids, whereas in the other populations only 7%– 8% accessions originated from hybridization events. This result indicates that the frequency of introgression depends not only on the overlap in flowering time, but also on other external influences such as the occurrence of nearby cultivars or the tree density in the population (Kramer et al., 2008; Reim et al., 2017).

### 4.3 Gene flow between the *M. sylvestris* populations in Saxony

Fragmented and spatially isolated populations of rare species such as *M. sylvestris* are often expected to have reduced genetic diversity and strong genetic structure because of a restricted gene flow (Bacles & Jump, 2011; Kramer et al., 2008; Pierce et al., 2017). However, despite the fragmented distribution of *M. sylvestris* in the study area, the results of our study indicate a high genetic diversity and a weak genetic structure between the single populations. Other studies also documented that particularly long‐lived plants such as *M. sylvestris* can maintain connectivity even in highly fragmented populations through extensive gene flow via pollen and/or seeds (Feurtey et al., 2017; Gonzalez‐Varo et al., 2019; Lowe et al., 2016; Ozawa et al., 2013; Plue & Cousins, 2018; Schnitzler et al., 2014; Wang et al., 2014). The comparison of nuclear and chloroplast data allows the separation of the impact of pollen and seed‐mediated gene flow between populations (Ennos, 1994). The *N_m_*‐value in our study indicated a high historical genetic exchange with a higher number of migrants by seeds (*N_m_* = 7.07) than by pollen (*N_m_* = 4.14.) between the *M. sylvestris* populations. Apple seeds are distributed by numerous wild animals such as mammals or birds, feed on the apple fruit. These animals travel over long distances and probably accounting for the large apple‐dispersal capacities (Schnitzler et al., 2014). In addition, humans dispersed wild apple fruits over long distances in recent times (Spengler, 2019). As a result, in the past genetic exchange by seeds between population occurred and even one single hybridization event retained within a lineage because the chloroplast genome is uniparental inherited (Currat et al., 2008).

Additionally, wild apple populations probably compensated their spatial isolation by higher pollen dispersal distances. Single located individuals can act as so‐called stepping stones and bridge larger distances between groups of trees (Albaladejo et al., 2012; Kramer et al., 2008). Thus, metapopulations could maintained their genetic exchange and high population genetic diversity. However, high pollen flow distances carry the risk of increased hybridization events between *M. sylvestris* and *M*. × *domestica* and may have negative effects on the maintenance of pure *M. sylvestris* individuals. Even if only less introgressed wild apples were observed in our study, the implementation of sustainable conservation strategies seems to be necessary for a long‐term preservation of this rare species.

## 5 CONCLUSIONS FOR CONSERVATION MEASURES

The clear genetic differentiation between *M. sylvestris* and *M*. ×*domestica* indicated a low gene flow between both species and the existence of pure *M. sylvestris* individuals in our study area. Our study has also shown that the genetic diversity of *M. sylvestris* is still high due to a high gene flow between the single populations. Nevertheless, introgression of *M. ×domestica* in wild apples takes place and endangers the preservation of the pure *M. sylvestris* individuals. Furthermore, most wild apples are located in unprotected areas and a reduction of the remaining population can be expected. Over time, this would lead to a loss of genetic diversity in *M. sylvestris* populations.

Therefore, the protection of existing *M. sylvestris* populations and implementation of preservations measures (e.g., replanting of young trees) are recommend. Particularly, the reintroduction of young trees and the increase of number of trees within the natural populations are recommended to reduce the risk of hybridization. An additional conservation of pure *M. sylvestris* accessions ex situ can be also meaningful. That can be implemented by the storage of seeds in a GenBank or the establishment of seed orchards.

## CONFLICT OF INTEREST

The authors declare that they have no conflict of interest.

## AUTHOR CONTRIBUTION

**Stefanie Reim:** Conceptualization (lead); Funding acquisition (equal); Investigation (lead); Project administration (equal); Writing‐original draft (lead). **Anke Proft:** Data curation (supporting); Investigation (equal); Project administration (equal); Validation (supporting). **Frank Lochschmidt:** Data curation (supporting); Investigation (supporting); Resources (supporting). **Monika Höfer:** Conceptualization (supporting); Resources (supporting); Validation (supporting).

## Supporting information

Fig S1

Table S1

Table S2

## Data Availability

The data that support the findings of this study are openly available in Dryad at https://doi.org/10.5061/dryad.g79cnp5n2
