# Supplementary material for: Genetic integrity is still maintained in natural populations of the indigenous wild apple species Malus sylvestris (Mill.) in Saxony as demonstrated with nuclear SSR and chloroplast DNA markers
Source: Ecol Evol. 2020 Sep 28;10(20):11798–809. doi: 10.1002/ece3.6818 (PMC7593173; doi:10.1002/ece3.6818)
Supplement: Supplementary file 1 — Fig S1 [file ECE3-10-11798-s001.docx]

*
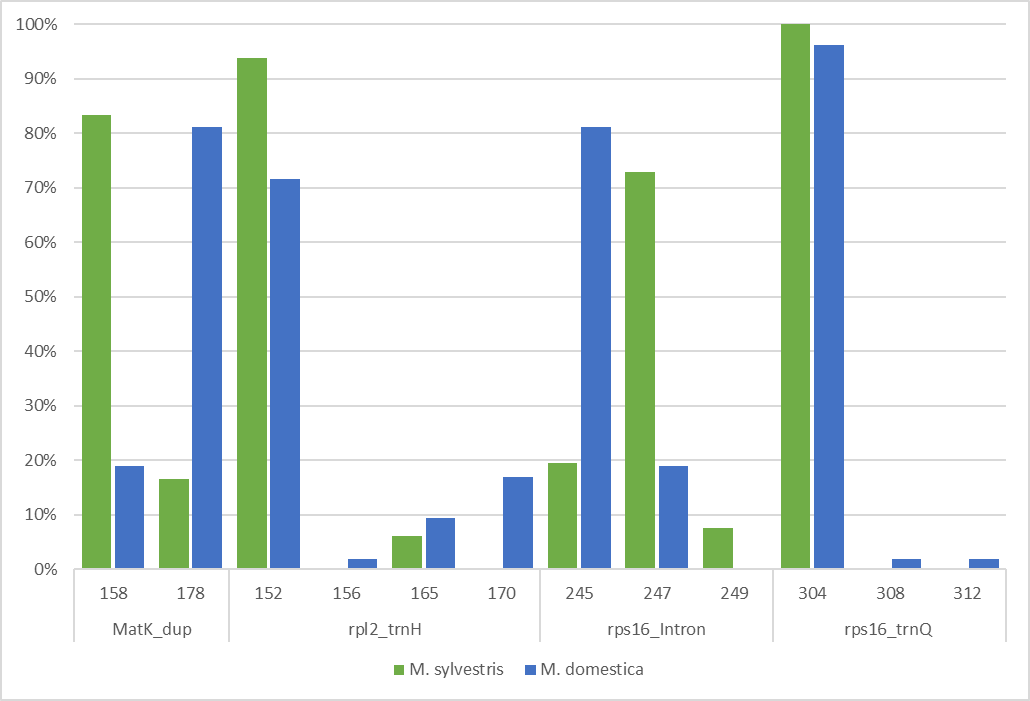
*

Supplement Figure 1: Allele frequency after amplification with the cpDNA markers *matk_dup, rpl2_trnH*, *rps16_Intron* and *rps16_trnQ* for the *M. sylvestris* and *M. ×domestica* samples*.*
