# Supplementary material for: Genetic integrity is still maintained in natural populations of the indigenous wild apple species Malus sylvestris (Mill.) in Saxony as demonstrated with nuclear SSR and chloroplast DNA markers
Source: Ecol Evol. 2020 Sep 28;10(20):11798–809. doi: 10.1002/ece3.6818 (PMC7593173; doi:10.1002/ece3.6818)
Supplement: Supplementary file 3 — Table S2 [file ECE3-10-11798-s003.docx]

Supplement Table 2: Allele combination of each haplotype based on the four different chloroplast markers

| Haplotype | *matK_dup* | *rpl2_trnH* | *rps16_Intron* | *rps16_trnQ* |
| --- | --- | --- | --- | --- |
| H1 | 158 | 152 | 245 | 304 |
| H2 | 158 | 152 | 247 | 304 |
| H3 | 158 | 152 | 249 | 304 |
| H4 | 158 | 156 | 247 | 308 |
| H5 | 158 | 165 | 245 | 304 |
| H6 | 158 | 165 | 245 | 312 |
| H7 | 158 | 165 | 247 | 304 |
| H8 | 158 | 165 | 249 | 304 |
| H9 | 158 | 170 | 245 | 304 |
| H10 | 158 | 170 | 247 | 304 |
| H11 | 178 | 152 | 245 | 304 |
| H12 | 178 | 152 | 247 | 304 |
| H13 | 178 | 152 | 249 | 304 |
| H14 | 178 | 170 | 245 | 304 |
| H15 | 178 | 170 | 247 | 304 |
